# Botulinum toxin A treats nail psoriasis but at what cost? Reply to “Botulinum toxin injection shows promise in nail psoriasis: A comparative randomized controlled trial”

**DOI:** 10.1016/j.jdin.2024.07.004

**Published:** 2024-08-25

**Authors:** Jose W. Ricardo, Shari R. Lipner

**Affiliations:** Department of Dermatology, Weill Cornell Medicine, New York, New York

**Keywords:** botulinum toxin A, clinical trial, intralesional, nails, psoriasis, triamcinolone acetonide

*To the Editor:* We read with interest Juntongjin et al,’s^1^ article on botulinum toxin A (BoNT-A) injections for treating nail psoriasis (NP). In an intra-subject trial, 64 psoriatic fingernails (16 patients) were randomized into 4 groups: intralesional abobotulinum toxin A (Abo-BoNT-A) (baseline), intralesional 10 mg/ml triamcinolone acetonide (TAC) (weeks 0, 8), topical vitamin D/steroid combination (16 weeks), or no treatment. By week 16, all groups showed reduction in target Nail Psoriasis Severity Index (NAPSI) scores (*P* < .1), with no significant difference among groups at any time point; the Abo-BoNT-A group continued to improve, while the TAC group remained stable at week 24. We commend the authors’ efforts in exploring novel NP treatment approaches. In this letter, we discuss potential challenges in adopting intralesional BoNT-A into standard NP care.

Juntongjin et al^1^ showed that a single Abo-BoNT-A injection provides a long-lasting effect in treating NP, comparable to multiple TAC injections. However, BoNT-A injection for NP treatment may not be cost-effective. As of 2024, one unit of Abo-BoNT-A (Dysport) costs approximately $1.9 ($559.80/300-unit vial, Galderma) in the United States. Juntongjin et al^1^ utilized up to 4 Abo-BoNT-A injections/nail using 7.5 units/site, which would cost $57/nail and $570/ten fingernails per treatment. Considering the similar efficacy of Abo-BoNT-A to intralesional TAC or topical vitamin D/steroid for NP, the higher cost is likely not justified. In our experience, up to 10 fingernails could be successfully treated with intralesional TAC, which is well-tolerated using ethyl chloride refrigerant spray, deep-breathing technique,^2^ and distraction. With a 5-ml vial of 10 mg/ml TAC priced at $14 (McKesson, 2024), treating 10 fingernails with a 0.1 ml proximal nailfold injection each of a 2.5 mg/ml TAC dilution uses 0.25 ml of the original vial, costing $0.7/treatment.

Juntongjin et al^1^ reported that Abo-BoNT-A toxin was particularly effective for nail bed (NB) psoriasis, possibly because the NB is highly vascularized and innervated. The authors used a digital nerve block with 2% lidocaine HCL and topical 5% lidocaine/prilocaine, followed by 2 nail matrix and 2 NB injections per digit. This protocol appears unnecessarily invasive, reflected in an average pain rating of 5/10. In our recent intra-subject, randomized controlled trial with 10 patients (73 fingernails) assessing intralesional TAC concentrations for NP, a single matrix injection of 2.5 mg/ml TAC every ∼6 weeks for 3 treatments reduced NAPSI bed scores by 40% at 24 weeks (*P* < .001).^3^ Improvement in NB NAPSI scores could be attributed to low TAC concentrations diffusing from the matrix injection into the NB, thus eliminating the need for multiple NB injections.

Intralesional therapy holds a unique place in the NP’s treatment armamentarium due to the nail plate having low penetrability. While intralesional Abo-BoNT-A might be effective for treating NP, its cost—approximately 814 times higher than intralesional TAC per treatment—and the need for multiple NB injections are significant barriers to implementation in clinical practice. Larger studies with extended follow-up are necessary to evaluate the duration of action across different NP treatments. Until then, we recommend that more cost-effective intralesional alternatives, including TAC and methotrexate,^4^ are used as first-line.

## Conflicts of interest

Dr Lipner has served as a consultant for Ortho Dermatologics, Eli Lilly, BelleTorus Corporation, and Moberg Pharmaceuticals. Dr Ricardo has no conflicts of interest to declare.
